# Effects of Different Drying Processes on Bioactive Components, Volatile Compounds, and In Vitro Inhibition of Starch Digestion in Mulberry Leaf Extracts

**DOI:** 10.3390/foods14060998

**Published:** 2025-03-14

**Authors:** Haizhi Li, Guoyu Liu, Yifeng Liu, Peng Yuan, Shiwei Liu, Mengqing Yan, Yan Zou, Haotian Wang, Tianyu Zhang, Shenglin Duan, Chao Ma

**Affiliations:** 1 Beijing Key Laboratory of Forest Food Processing and Safety, College of Biological Science and Technology, Beijing Forestry University, Beijing 100083, China; li_haizhi@163.com; 2 Beijing Key Laboratory of the Innovative Development of Functional Staple and Nutritional Intervention for Chronic Disease, China National Research Institute of Food and Fermentation Industries, Beijing 100015, China; lgy17091037820@163.com (G.L.); liuyif2020@163.com (Y.L.); 13401170514@163.com (P.Y.); 15101156761@163.com (S.L.); ymq20000818@163.com (M.Y.); zouyanyan211@163.com (Y.Z.); wht1275930132@126.com (H.W.); zhangtianyu990622@163.com (T.Z.); 3 State Key Laboratory of Efficient Production of Forest Resources, Beijing Forestry University, Beijing 100083, China

**Keywords:** mulberry leaf, ethanol extract, microstructure, GC-IMS, inhibitory effect

## Abstract

The significant demand for medicinal plants with special efficacy has prompted us to adopt appropriate processing methods to enhance the nutritional quality and flavor of raw materials. This study evaluated the impacts of freeze-drying (FD), hot-air drying (HAD), and spray drying (SD) on the bioactive compounds, flavor characteristics, and inhibition of starch digestion in mulberry leaf ethanol extract (MLE). Results indicated that FDMLE exhibited the highest total alkaloids content (TAC: 0.14 ± 0.02 mg/g) and total flavonoid content (TFC: 19.32 ± 0.58 mg/g), along with significant inhibitory effects on starch hydrolysis at 180 min (starch hydrolysis rate <50%). The microstructure of HADMLE was closest to that of the mulberry leaf powder (ML), but SD better preserved the color of ML (Δ*E* = 1.55 ± 0.04). Combined with the electronic nose and gas chromatography-ion mobility spectrometry (GC-IMS) found HAD processing facilitated the conversion of flavor precursors in ML into Ethyl formate, rose oxide, and (Z)-3-hexenol (M). SDMLE contained higher levels of pentanal, (E)-2-hexenal (D), (E)-2-pentanone, 3-Methyl-2-butenal (D), ethyl butyrate, and 1-penten-3-one (D). FDMLE exhibited the highest diversity of novel volatile compounds (VOCs), with 18 newly identified species. In conclusion, FD is a potential method to effectively reduce the degradation of quality and efficacy of MLE during the drying process.

## 1. Introduction

Mulberry leaves (*Morus alba* L.), belonging to the Moraceae family and the genus Morus, are a medicinal plant extensively utilized in traditional medicine [1]. Mulberry leave (ML) is abundant in bioactive compounds, including flavonoids, alkaloids, polyphenols, sterols, as well as various inorganic trace elements and vitamins. Among these, flavonoids, alkaloids, and polyphenols have a strong correlation with diabetes [2]. The mechanisms of blood glucose reduction primarily involve inhibiting intestinal α-glucosidase activity, regulating lipid metabolism, protecting pancreatic cells, reducing insulin resistance, accelerating glucose uptake by target tissues, and improving oxidative stress levels in the body [3,4]. Han et al., utilizing a hyperglycemic mouse model, identified flavonoids and alkaloids as the key components responsible for the reduction of blood glucose [5]. Furthermore, the synergistic interactions among the various components of MLs may enhance their efficacy in alleviating diabetic syndromes [6]. With the increasing demand for natural, nutritious, and healthy foods among consumers, MLs have garnered increasing attention for their distinctive health advantages. However, the active ingredients in ML are prone to degradation, and its color tends to oxidize and deteriorate, resulting in unstable efficacy and hindering the healthy development of the ML industry. Therefore, it is imperative to adopt appropriate methods for the deep processing of ML.

Studies have demonstrated that drying methods are critical in the processing of bioactive extracts, as they effectively minimize nutrient degradation, inhibit spoilage microorganisms, retard enzymatic activity, and reduce moisture-mediated reactions [7,8]. Common drying methods include freeze-drying (FD), hot-air drying (HAD), and spray drying (SD), each of which has different advantages in maintaining the structural and functional activity. FD involves complex processes including freezing, sublimation, desorption, and rehydration, all of which may impact the quality of the final product. However, when performed under low-temperature and vacuum conditions, this technique avoids the phase transition of water into the liquid phase, thereby minimizing thermal damage to samples. This approach effectively preserves biological activity and complex structures, prevents thermal degradation, and consequently, enhances product yield [9,10]. Currently, FD technology is also widely used in industrial production. This technology not only helps preserve the medicinal efficacy or chemical activity of products but also extends their shelf life. Additionally, it reduces losses during subsequent processing or packaging stages, thereby lowering overall production costs. HAD is a rapid and cost-effective method that allows for precise control over process parameters such as temperature, drying duration, and air velocity. However, its prolonged drying durations and elevated temperatures may lead to thermal degradation and compromise product quality [11]. SD is a method that atomizes liquid feed into fine droplets and achieves rapid moisture evaporation under a stream of hot air, ultimately yielding a powdered product. This technique is particularly advantageous due to its high drying efficiency, ability to effectively preserve heat-sensitive components, and capacity to maintain the bioactivity or flavor profiles of the sample [12]. During the drying process, product characteristics such as active constituents, hypoglycemic efficacy, color attributes, and flavor profiles may undergo alterations [13,14]. The presence of undesirable flavors, primarily characterized by a “grassy” taste in raw materials of MLs, poses a significant constraint on the future development of ML. MLs contain volatile compounds (VOCs) such as alcohols, ketones, and unsaturated fatty acids like linoleic acid, which impart fruity and cheesy flavors [15]. The scarcity of research on identifying flavor compounds in mulberry leaf extracts (MLEs) prepared using different drying methods has significantly hindered the development of flavor improvement technologies and the production of high-quality ML-based food products.

The hypoglycemic efficacy of MLE has been well-documented [16,17,18]. However, research on the impact of different drying methods on the flavor profiles of MLE remains limited. Therefore, in this study, total alkaloids (TA) and total flavonoids (TF) from ML were extracted by the ethanol method, aiming to investigate the effects of HAD, SD, and FD on the active components, microstructural features, color, and VOCs. In addition, the inhibitory effects of different drying methods on starch digestion were investigated to screen for the optimal drying method of MLE that possesses desirable flavor characteristics and strong anti-starch digestive activity. These findings provide theoretical foundations for selecting drying strategies to maximize MLE product quality.

## 2. Materials and Methods

### 2.1. Materials

Dried mulberry leaves were purchased from a mulberry leaf collector in Guangzhou City, Guangdong Province, China. Corn starch was acquired from Hebei Tianren Jiaye Agricultural Products Co., Ltd. (Hebei, China). Potassium borate solution, 9-Fluorenylmethyl chloroformate (FMOC-Cl), and 1-deoxynojirimycin (DNJ) were the standard products used in this study and were acquired from Shanghaiyuan ye Bio-Technology Co., Ltd. (Shanghai, China). Chromatographic grade acetonitrile and acetic acid were purchased from Merck (Darmstadt, Germany). Unless otherwise specified, all other chemicals and reagents were obtained from Shanghai Macklin Biochemical Co., Ltd. (Shanghai, China).

### 2.2. Preparation of Samples Using Different Drying Methods

The extraction process was performed using 5 g of ML with 60% (*v*/*v*) ethanol as the extraction solvent. The extraction conditions were optimized as follows: a solvent-to-sample ratio of 30:1 (*v*/*w*), pH adjusted to 6.0, and an extraction duration of 1.5 h, and two consecutive extraction cycles as the baseline condition. The extraction yield of TF and TA under different extraction times, extraction frequencies, temperatures, solid-to-liquid ratios, ethanol concentrations, and pH values was investigated to determine the optimal extraction conditions for preparing MLE. The collected supernatant was concentrated using a rotary evaporator (HB10, IKA Works Guangzhou, China). The concentrated samples were then dried using the following methods.

FD: MLE was frozen in a vacuum freeze dryer (LGJ-18, Beijing Songyuanhuaxing Technology Develop Co., Ltd., Beijing, China) at −80 °C for 36 h. Removed the sample when the instrument temperature reached room temperature.

HAD: MLE was put into the electric blast dryer (Shanghai YIHENG Technical Co., Ltd., Shanghai, China) at 80 °C for 72 h. The MLE was weighed every 4 h until it reached a constant weight.

SD: MLE was mixed by an electric stirrer (D2010W, Shanghai Meiyingpu Instrument Manufacturing Co., Ltd., Shanghai, China), and finally dried in a spray dryer (B-290, BUCHI Labortechnik AG, Flawil, Switzerland) under the conditions of inlet air temperature 150 °C.

### 2.3. Determination of TFC and TAC

We used rutin as a reference standard to determine TFC [19]. Briefly, a suitable volume of the 1 mL sample was transferred to a 5 mL centrifuge tube, to which 0.15 mL of 5% sodium nitrite solution was added. Subsequently, 0.15 mL of 10% aluminum nitrate solution and 2 mL of 4% sodium hydroxide solution were added. After vortexing for 15 min, the absorbance was then measured at 510 nm using a microplate reader (SpectraM ^®^ i3, Molecular Devices, Sunnyvale, CA, USA). The TAC was measured according to the method described by Yu et al. [20]. TAC detection was based on DNJ standard, with a sample of 100 µL. We added 175 µL of 0.4 mol/L potassium borate solution and 250 µL of 5 mmol/L derivative reagent FMOC-Cl acetonitrile solution sequentially. This was reacted at 25° C for 25 min, then we added 100 µL of 0.1 mol/L glycine solution and reacted for 20 min. We added 75 µL of 1% acetic acid solution and 300 µL of deionized water by volume fraction. We used ultra-high liquid phase equipped with PDA detector (H-Class, Waters Corporation, Milford, CT, USA) and an ACQUITY UPLC C18 column (2.1 mm × 50 mm, Waters Corporation, Milford, CT, USA). The mobile phase was acetonitrile (A): 0.1% acetic acid (B) = 30:70, with a flow rate of 1.0 mL/min and an injection volume of 10 µL. The moisture content (MC) of the sample was measured using a rapid moisture analyzer (MA150, Sartorius AG, Göttingen, Germany).

### 2.4. Scanning Electron Microscopy

Sample morphology was examined using a Phenom ProX scanning electron microscope (SEM, Delmic B.V., Delft, The Netherlands) according to Kim et al. [21], with an acceleration voltage of 15 kV and 500× magnification.

### 2.5. Color Measurement

The color was measured using an automatic color difference meter (SE6000, Shanghai Shouli Industrial Co., Ltd., Shanghai, China). Calibration was performed using standard black and white tiles, followed by placing the sample in a transparent colorimetric dish for testing. It was expressed using the color difference value (Δ*E*) [22]:

$$\Delta E=\sqrt{\Delta L^{2}+\Delta a^{2}+\Delta b^{2}}$$

where L* is the brightness index, a* and b* are indicators of the color chroma, and Δ*L*, Δ*a*, and Δ*b* are the difference in the *L*, *a*, and *b* values of ML before and after drying.

### 2.6. Electronic Nose Analysis

Approximately 2 g of MLE samples prepared by different drying methods were weighed and placed into 20 mL headspace vials [23]. The vials were sealed, and the headspace gas was extracted for detection and analysis using an electronic nose (FOX4000, Alpha M.O.S, Toulouse, France). The instrument is equipped with 17 sensors, each with specific performance characteristics: LY2/LG: fluorine, chlorine, nitrogen, and oxygen compounds. LY2/G: ammonia, amine compounds and carbon oxides. LY2/gCTL: hydrogen sulfide. LY2/gCT: propane and alkanes. T30/1: organic compounds. P10/1: hydrocarbons. P10/2: methane. P40/1: fluorine. T70/2: toluene and xylene. P30/1: hydrocarbon combustion products. P40/2: chlorine. T40/2: chlorine. T40/1: fluorine. TA/2, LY2/AA, PA/2, and P30/2: alcohol compound. The measurement parameters were set as follows: collection time of 120 s, flow rate of 150 mL/min, injection volume of 2000 µL, cleaning time of 120 s, incubation time of 180 s, incubation temperature of 60 °C, and injection flow rate of 1000 µL/s. Each sample groups were subjected to three parallel tests.

### 2.7. Determination of Volatile Compounds

The VOCs in samples subjected to different drying methods were analyzed using a FlavorSpec^®^ GC-IMS (gas chromatography-ion mobility spectrometry, G.A.S., Berlin, Germany) with a WAX quartz capillary column (30 m × 0.53 mm, 1 μm; Restek Corporation, Bellefonte, PA, USA) according to the method described by Xu et al. [24].

Briefly, 2.0 g of each MLE sample was transferred into a 20 mL glass vial and incubated at 60 °C for 15 min. The headspace gas (100 µL) was automatically injected into the syringe with the syringe temperature set to 85 °C, incubation shaking speed at 500 r/min, and high-purity N_2_ as the carrier gas.

For GC analysis, the column temperature was maintained at 60 °C, and the carrier gas flow rate program was set as follows: initial 2.0 mL/min, held for 2 min, linearly increased to 10 mL/min over 10 min, then to 100 mL/min over 20 min, with a total run time of 30 min.

The IMS analysis conditions included: drift tube length of 98 mm, linear voltage in the tube of 500 V/cm, drift tube temperature of 45 °C, drift gas of high-purity N_2_, drift gas flow rate of 150 mL/min, radiation source of β-ray (^3^H), and operation in positive ion mode. The GC-IMS detection results are presented as three-dimensional signal absorption peak maps. Qualitative analysis of substances in the two-dimensional spectra was conducted using the instrument’s accompanying Laboratory Analytical Viewer software, integrated with the built-in NIST gas-phase retention index database and the G.A.S. IMS migration time database. Differential comparative analysis of volatile compounds was performed using the Reporter and Gallery Plot plugins.

### 2.8. Determination of In Vitro Digestibility

The hydrolysis rate of corn starch in the presence of MLE prepared by different drying methods was evaluated according to the method described by Ding et al. [25]. Briefly, 100 mg of MLE was thoroughly mixed with 200 mg of corn starch. The mixture underwent simulated oral and gastric digestion phases, followed by in vitro simulated intestinal digestion. During the intestinal digestion phase, 500 µL of the digestive fluid was collected at 0, 20, 40, 60, 90, 120, 150, and 180 min and transferred into a 10 mL centrifuge tube containing 4.5 mL of anhydrous ethanol to inactivate the enzymes. After enzyme inactivation, the mixture was centrifuged at 5000 r/min for 5 min to obtain the supernatant. The glucose concentration in the supernatant was measured using a glucose assay kit (F006-1-1, Nanjing Jiancheng Bioengineering Institute, Nanjing, China).

### 2.9. Statistical Analysis

The data presented in the tables and figures were obtained from a minimum of three independent measurements. For each measurement, the mean value and standard error were calculated. The statistical analysis was performed by using the software Origin Pro 2024 software (OriginLab, Northampton, PA, USA). The significant differences were carried out using one-way ANOVA (Tukey’s multiple range tests), and *p* < 0.05 was considered to indicate statistically significant differences among the components.

## 3. Results and Discussion

### 3.1. Effects of Extraction Conditions on the Yield of TF and TA from ML

**Figure 1:**
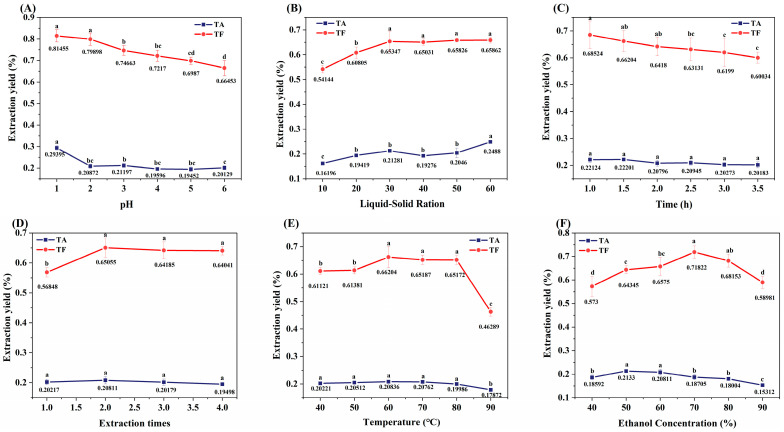
Effects of extraction conditions on extraction yield of flavonoids and alkaloids from ML. Different letters of a–d indicate a significant difference (*p* < 0.05). (**A**) represents the effect of pH on the extraction yield; (**B**) represents the influence of the ratio of liquid-solid on the yield; (**C**) represents the influence of time on extraction yield; (**D**) represents the influence of extraction times on extraction yield; (**E**) represents the influence of temperature on yield; (**F**) represents the effect of ethanol concentration on the yield.

The bioactive components of ML were extracted using acidified ethanol, with the yield of TA and TF serving as evaluation indices (Figure 1). The extraction efficiency exhibited significant pH dependence, as the yields of both TA and TF decreased markedly when the pH exceeded 2.0 (Figure 1A). This phenomenon can be attributed to the neutralization reaction between H^+^ and alkaloids present in the extraction system. The solid-to-liquid ratio demonstrated a substantial influence on extraction efficiency. At a ratio of 1:30, the TF yield showed a significant increase followed by stabilization, while the TA yield required a higher ratio of 1:60 to achieve significant enhancement (*p* < 0.05) (Figure 1B). Temporal analysis revealed that extraction time had no significant impact on TA yield (*p* > 0.05), whereas prolonged extraction (>3 h) led to a significant reduction in TF yield (*p* < 0.05) (Figure 1C). Multiple extraction cycles analysis indicated that more than two extraction cycles did not significantly improve TF and TA yield (*p* > 0.05) (Figure 1D). Temperature had a minor effect on TA, but when the temperature exceeded 80 °C, the yield of TF significantly decreased (*p* < 0.05) (Figure 1E), which may be attributed to the thermal instability of flavonoid cyclic structures [26]. Increasing ethanol concentration promotes TF extraction [27], but the yields of both TA and TF initially increased and then decreased with rising ethanol concentration (Figure 1F). The optimal extraction conditions for the simultaneous recovery of TA and TF using acidified ethanol were determined as follows: pH 1.0, extraction temperature of 60 °C, extraction duration of 1 h, solid-to-liquid ratio of 1:30 (*w*/*v*), two consecutive extraction cycles, and ethanol concentration of 60% (*v*/*v*).

### 3.2. Determination of Bioactive Compounds

The overall differences in WC, TAC, and TFC among MLE processed using different drying methods were significant (Table 1). The study found that increasing temperature and the presence of oxygen negatively affect the stability of bioactive components such as ketones. The drying method had a minimal impact on TAC. However, the TFC ranged from 4.89 to 19.32 mg RE/g DW, with the TFC in MLE prepared by FD being significantly higher than that in the other two methods (*p* < 0.05). This indicated that during the FD process, the samples are maintained under vacuum conditions with extremely low oxygen levels, which effectively inhibits the oxidation of flavonoids [28]. Compared to FD method, the HAD method process required higher temperatures and time to promote water evaporation, ultimately leading to a reduction in TFC in the samples. Fan et al. [29] reported similar results that FD Taihang chrysanthemum exhibited a significantly higher TFC (52.863 ± 0.653 mg GAE/g) compared to HAD samples (5.905 ± 0.512 mg GAE/g).

### 3.3. Morphology Characterization

**Figure 2:**
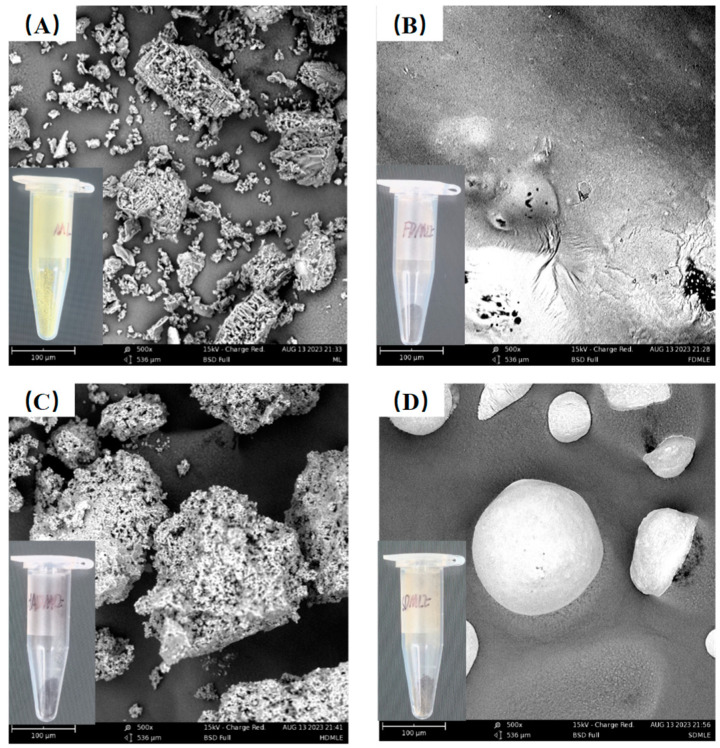
The SEM morphology of MLE by different drying methods. The figure marked with (**A**–**D**) shows the morphology of ML, FDMLE, HADMLE, and SDMLE, respectively.

Extraction methods impurities are key factors influencing the SEM structure of MLE. MLEs prepared using different drying techniques display distinct morphological features under SEM analysis (Figure 2). In samples prepared by HAD and SD methods, moisture diffuses from the inside out [30]. The elevated temperatures used in these methods can adversely affect MLE, causing tissue disintegration. Specifically, MLE obtained via the HAD method appear as aggregated, porous blocks, resembling the structure of ML. In contrast, MLE produced by the SD method exhibit unevenly sized spherical particles with smooth surfaces, enhancing food lubricity and texture [31]. For FDMLE, moisture forms ice crystals under vacuum and low-temperature conditions, which then directly sublimate, resulting in a more compact structure and smoother sheet-like appearance.

### 3.4. Color

Color is one of the important indicators affecting the quality characteristics of ML, and its chromaticity influences consumer preferences. Table 2 showed the chromaticity of MLE under different drying methods. The a* and b* values represent red/green and yellow/blue, respectively, the L* value represents brightness, and the Δ*E* value represents the overall degree of visual difference between ML and MLE prepared by different drying methods. The Δ*E* value is positively correlated with browning intensity, indicating that higher Δ*E* values in dried samples corresponded to more pronounced color differences between dried and fresh samples [32]. Compared to ML, the L*, a*, and b* values of MLE prepared by different drying methods showed certain variations, which may be attributed to changes in anthocyanin content due to significant moisture loss [33]. The a* and b* values of SDMLE showed no significant difference from those of ML (*p* > 0.05), and the Δ*E* value was the lowest, indicating that SDMLE better preserved the color of ML. This may be because the high temperature during SD promotes the binding of polyphenolic compounds in ML with macromolecular substances such as proteins, forming stable complexes that enhance the stability and chromaticity of the extract [34]. The L*, b*, and Δ*E* values of HADMLE were significantly higher than those of MLE prepared by other drying methods (*p* < 0.05), possibly due to prolonged drying time leading to some degree of browning on the sample surface. After FD, the L*, b*, and a* values of MLE were moderate, likely because the low-temperature environment provided some protection to the sample’s chromaticity [35]. Huang et al. [36] investigated the effects of different drying methods on browning of *Choerospondias axillaris* fruits. They found that FD samples exhibited a lower browning degree, with Δ*E* values (14.53 ± 3.97) significantly lower than those of HAD samples (Δ*E* = 64.78 ± 5.11). This aligns with the findings presented in this research.

### 3.5. Volatile Compounds

#### 3.5.1. Electronic Nose Analysis

**Figure 3:**
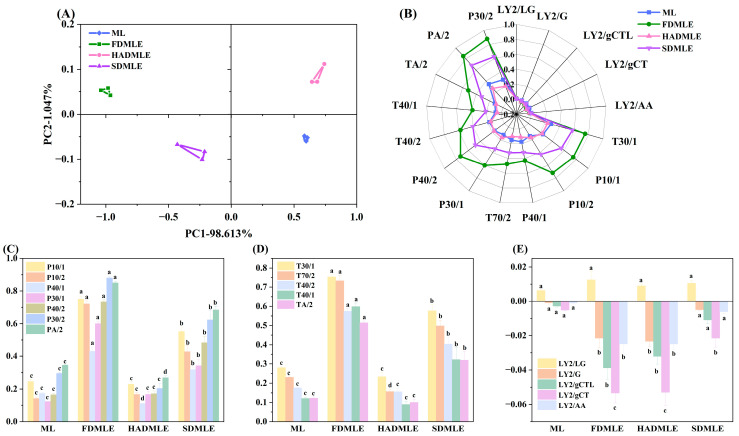
(**A**) Principal component analysis (PCA) plot of MLE prepared by different drying methods using an electronic nose. (**B**) Radar chart. (**C**) Changes in the signal responses of electronic nose sensors P10/1, P10/2, P40/1, P30/1, P40/2, P30/2, and PA/2. (**D**) Changes in the signal responses of electronic nose sensors T30/1, T70/2, T40/2, T40/1, and TA/2. (**E**) Changes in the signal responses of electronic nose sensors LY2/LG, LY2/G, LY2/gCTL, LY2/gCT, and LY2/AA. Different letters of a–c indicate statistically significant differences (*p* < 0.05).

In the analysis of sample odor profiles using electronic nose technology, the contribution rate in principal component analysis (PCA) serves as a critical indicator of how effectively the principal components capture odor-related information. Generally, when the contribution rate of two principal components exceeds 70%, it indicates that the electronic nose can adequately capture sample odor information [37]. Figure 3A shows the PCA score plot of the odor of MLE prepared by three different drying methods. The total contribution rate of the first and second principal components reached 99.66%; it could be observed that samples prepared by different drying methods were distributed in different quadrants, indicating a noticeable distinction in their odor profiles.

Figure 3B,C–E display the electronic nose radar plots and sensor response values of MLE prepared by different drying methods, respectively. The response values of P30/2 and PA/2 sensors were higher, with substantial variation between samples, suggesting an increase in alcohol compound content after drying. This phenomenon may be attributed to the extraction method. Notably, the signal response value of FDMLE on P-type and T-type sensors was significantly higher than that of MLE prepared by other drying methods (*p* < 0.05). The results demonstrated that FD not only preserved ethanol-derived flavors during extraction, but also enhanced the levels of other VOCs. Both ML and MLE prepared by different drying methods exhibited low response values on the LY-type sensor, suggesting potential consumption of nitrogen- and oxygen-containing compounds during processing. Additionally, the LY-type sensor primarily detects odorants such as propane, butane, and hydrogen sulfide; it infers that these odor compounds are consumed at a rate higher than normal diffusion or oxidative metabolism due to the small size effect of the powder, resulting in very low responses for these odor substances [38].

#### 3.5.2. Comparative Analysis of Gas Chromatography-Ion Mobility Spectrometry

Most VOCs are formed during the drying process and the degradation of lipids and proteins [39]. Based on the results from the electronic nose, it was found that MLEs prepared using different drying methods exhibited distinct flavor profiles. To further analyze and compare the VOCs of MLEs prepared by various drying methods, gas chromatography-ion mobility spectrometry (GC-IMS) technology was employed.

**Figure 4:**
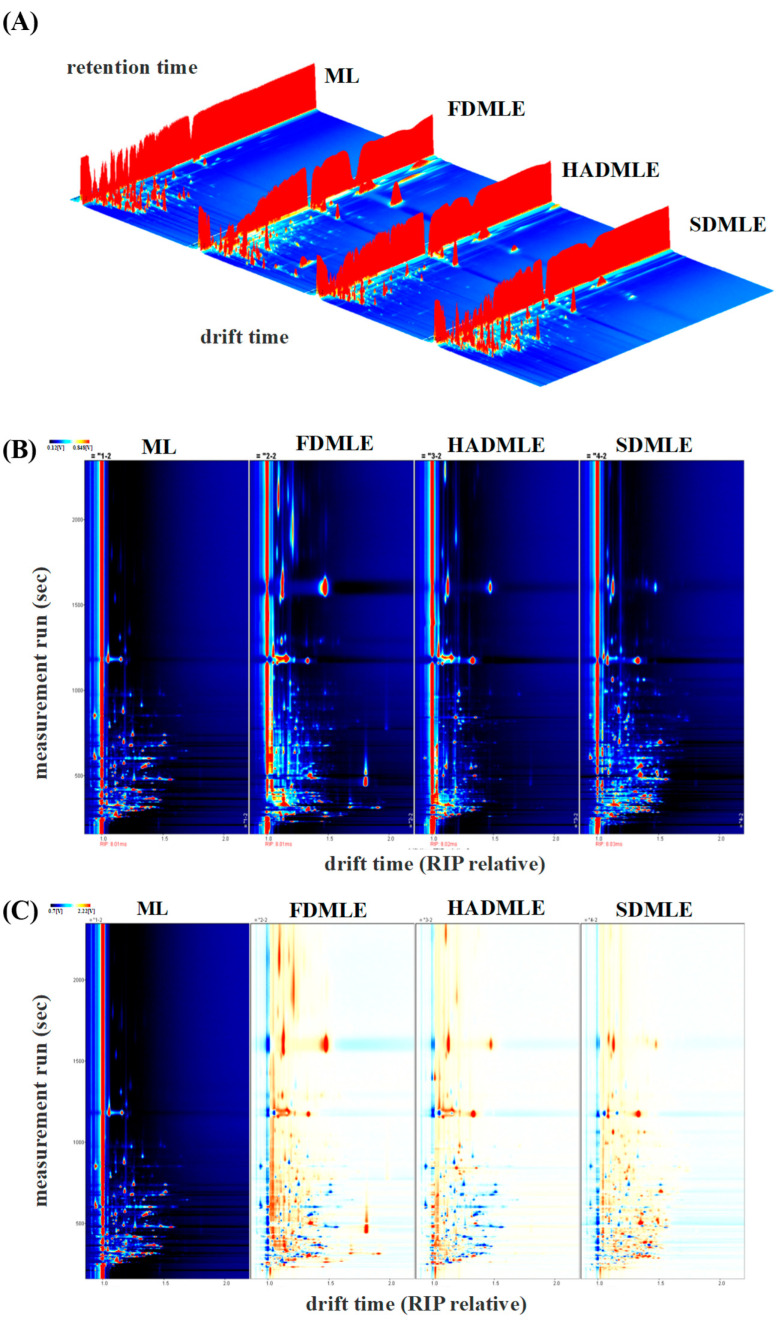
Three-dimensional GC-IMS spectra of VOCs in MLE prepared under different drying methods. (**A**) Original three-dimensional GC-IMS spectra. (**B**) Differential spectra of VOCs in MLE. (**C**) Qualitative analysis.

**Figure 5:**
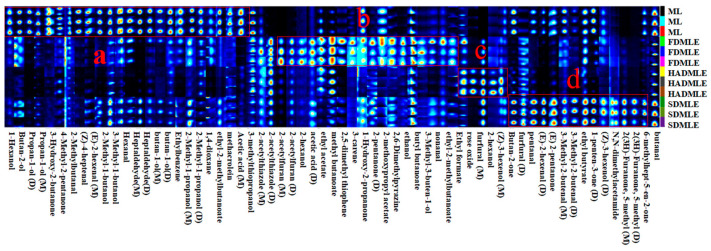
Fingerprint of volatile flavor substances in MLE prepared by different drying methods. a, b, c and d represent the special VOCs of ML, FDMLE, HADMLE and SDMLE, respectively.

The GC-IMS analyzed generated the three-dimensional spectrum (retention time, migration time, and peak intensity) (Figure 4A) and the vertical view (Figure 4B), difference diagram of sample (Figure 4C), and fingerprint (Gallery Plot) (Figure 5) of VOCs. In Figure 4B, the red vertical line at a migration time of 1.0 ms represents the reactive ion peak (RIP). Each point to the right of the RIP corresponds to a VOC detected in the MLE sample. Most signals appeared within a retention time range of 100–800 s and a drift time range of 1.0–1.5 s. The color of the points indicates the concentration of the substances, with blue representing lower concentrations and red representing higher concentrations [40]. The number and position of red dots in the spectra varied significantly, indicating differences in the composition of flavor compounds in MLE prepared using different drying methods. Using ML as a reference, the signal peaks in ML were subtracted from the other spectra to obtain the difference plots (Figure 4C). The deeper the blue or red color, the greater the difference observed [41].

To further compare the differences in VOCs of MLE under different drying methods, a comparative analysis of the VOCs fingerprint profiles was conducted (Figure 5). Each row represents a treatment group, with higher VOCs content indicated by brighter and redder colors. The figure marked four regions (a, b, c, and d), indicating significant differences in VOCs among the different drying methods. The following preliminary conclusions can be drawn from combining Table 3 with Figure 5. After drying, some of the original volatile substances of ML were lost, while new volatile substances were released. Based on the fingerprint profiles, a total of 63 VOCs, including monomers and dimers, were detected. These VOCs were primarily classified into four categories: 15 aldehydes, 16 alcohols, 8 ketones, and 8 esters. Compounds such as ethers, aromatic hydrocarbons, amides, and thiols were present in trace amounts. Among these, aldehydes were the main components contributing to the aroma of ML and their extracts. Aldehydes served as key indicators of drying odors, as they were associated with protein hydrolysis and amino acid degradation [42]. Ketone compounds, on the other hand, resulted from fatty acid or alkane degradation, Maillard reactions, and microbial esterification. At low concentrations, ketones impart flavors such as butter, blue cheese, and spiciness. Esters were formed by the condensation of alcohols and coenzyme A-activated acids [43]. Alcohols, generated through the oxidation of lipids and amino acids, contribute to the production of fatty aromas [44].

**Figure 6:**
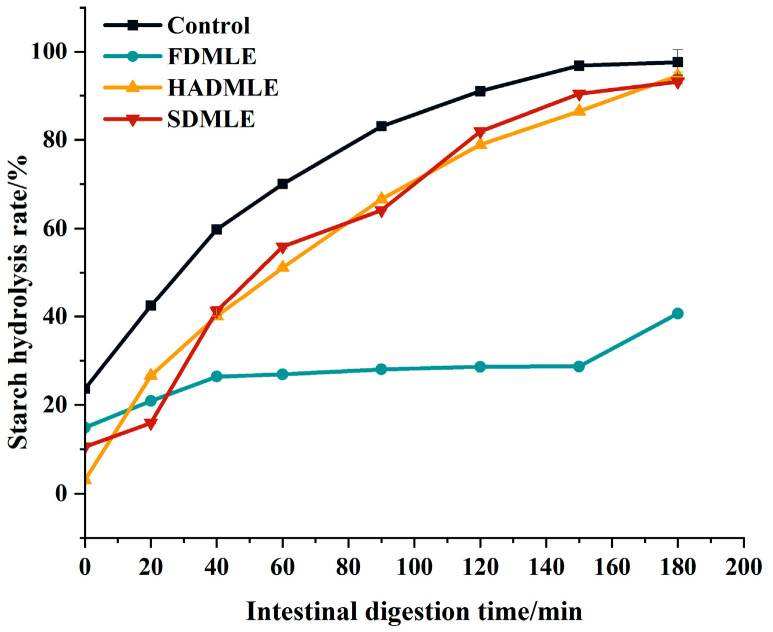
The impact of different drying methods on the starch hydrolysis rate of MLE. Different letters of a–c indicate a significant difference (*p* < 0.05).

The peak intensities of VOCs in the fingerprint spectrum indicated that the VOC content in ML undergoes significant changes after extraction. The volatiles of FD, HAD, and SD samples were different in their composition, while the FD samples contained more new volatile substances. In contrast to our findings, Bi et al. [45] proposed that SD samples contained more new volatile substances. Region a in Figure 6 highlights the characteristic VOCs of fresh ML, primarily comprising: 10 alcohols (1-Hexanol, Butan-2-ol, Propan-1-ol (D), Propan-1-ol (M), 2-Methyl-1-butanol, 3-Methyl-1-butanol (D), butan-1-ol (D), butan-1-ol (M), 2-Methyl-1-propanol (D), 2-Methyl-1-propanol (M)), 7 aldehydes (2-Methylbutanal, (Z)-4-heptenal, (E)-2-hexenal (M), Hexanal, Heptaldehyde (D), Heptaldehyde (M), methacrolein), 2 ketones (3-Hydroxy-2-butanone, 4-Methyl-2-pentanone), 1 aromatic hydrocarbon (Ethylbenzene), 1 heterocyclic compound (1,4-dioxane), and 1 ester (ethyl-2-methylbutanoate). These compounds were typical VOCs in ML, but their levels were greatly reduced or entirely eliminated after drying. Notably, aldehydes such as (Z)-4-heptenal and (E)-2-hexenal (M), which contributed to a grassy odor [46,47], showed a significant decrease in concentration after drying. Previous studies have emphasized the impact of drying temperature on alcohol and ester retention [48]. 3-Methyl-1-butanol was identified as the alcohol responsible for producing the desired flavor. High alcohol contents are desirable to obtain products with flowery notes. While drying enhanced certain flavor attributes, it also led to drawbacks, such as the reduction of 3-Hydroxy-2-butanone and 2-Methylbutanal, although ketones with creamy and fruity aromas could enhance the flavor of food [49].

In region b, a total of 5 ester compounds (ethyl acetate, methyl butanoate, 2-methoxypropyl acetate, butyl butanoate, ethyl-2-methyl butanoate), 2 furan compounds (2-acetylfuran (D), 2-acetylfuran (M)), 1 aldehyde compounds (nonanal), 2 ketone compounds (1-Hydroxy-2-propanone, 2-pentanone (D)), 3 alcohol compounds (2-hexanol, ethanol, 3-Methyl-3-buten-1-ol), 2 heterocyclic compounds (2,5-dimethyl thiophene, 2,6-Dimethylpyrazine), 1 carboxylic acid compound (acetic acid), 1 monoterpene compound (3-carene), and 1 thiazole compound (2-acetylthiazole (D)) were detected. The ethanol content increased in FD, likely attributed to the retention of alcohol-derived flavor compounds in MLE during FD processing. It was obvious that the ethanol content of MLE increased after drying on account of high proton affinity of ethanol. Ethanol plays a supporting role in enhancing the aroma of esters, making the fragrance more intense [50]. Ethyl acetate is responsible for the fruity scent [51]. Clearly, the content of ethyl acetate increased after FD, which helped to mask the putrid odor in the sample. This rise in ethyl acetate could result from interactions between alcohols and free fatty acids generated via lipid oxidation. Additionally, 3-carene, a monoterpene, contributed a distinct cedar-like aroma to MLE.

Although high-temperature processing reduces the content of most compounds, it can also generate unique flavors-imparting substances in dried products. For instance, alcohols are prone to oxidation and conversion into aldehydes or ketones upon heating, with higher temperatures resulting in greater conversion [50]. Consequently, aldehydes and ketones levels in SDMLE were higher than in FDMLE and HDMLE. The unique VOCs in HDMLE include Ethyl formate, rose oxide, and (Z)-3-hexenol (M). Ethyl formate contributes to the raspberry flavor, while HAD also imparted rose and citrus aromas to MLE. The unique aldehydes and ketones in SDMLE mainly included 3-Methyl-2-butenal (D), 3-Methyl-2-butenal (M), (E)-2-hexenal(D), pentanal, furfural (D), Butan-2-one, (E)-2-pentanone, 1-penten-3-one (D), 2(3H)-Furanone, 5-methyl (D), 2(3H)-Furanone, 5-methyl (M) and 6-methylhept-5-en-2-one. It has been reported that high concentrations of aldehydes and ketones contribute to improving the quality of cocoa, producing fruity and floral aromas. Previous studies have confirmed that during the dehydration process of *F. velutipes*, the content of aldehydes, alcohols, and some other volatiles increases [52]. Aldehydes, VOCs derived from lipids, fruits, and spices, were prominent in SDMLE. Pentanal and 3-Methyl-2-butenal were particularly abundant, with the former adding a fruity note to the overall flavor and the latter providing a bitter almond taste, enhancing the complexity of the overall aroma. However, Butan-2-one, pentanal, 3-Methyl-2-butenal (M), 1-penten-3-one (D), 6-methylhept-5-en-2-one, and other flavor compounds imparted a strong aroma of grass, nuts, and fat to SDMLE. At the same time, the acetic acid concentrations of HDMLE and SDMLE were significantly reduced, which indicated that the glycometabolic transformation of flavor substances could be effectively inhibited under this condition. Both HDMLE and SDMLE contained furfural, formed via a Maillard reaction between flavor precursors and amino acids in MLE under high temperatures. This reaction altered the overall flavor profile, resulting in the generation of new compounds with a roasted flavor [45].

### 3.6. Effect of Different Drying Methods on Inhibition of Starch Digestion of MLE

The extent to which the nutritional components in food exert their physiological functions within the body is primarily determined by the processes of human digestion and absorption. Simulating in vitro digestion processes enables relatively simplified and convenient investigation of bioactive substances in food. Through in vitro simulated digestion using enzymes commonly present in the human digestive tract (such as amylase, pepsin, and trypsin) applied to food matrices, macromolecular substances in food can be enzymatically hydrolyzed into smaller molecular entities. This enzymatic breakdown facilitates the release of active components from the food matrix. In vitro digestion models are widely used in food science research due to their convenience, repeatability, and cost-effectiveness compared to animal and human studies [53,54].

Therefore, while ensuring the flavor of MLE, we further evaluated the effects of MLE prepared by different drying methods on corn starch digestion using an in vitro simulated gastrointestinal digestion experiment, as shown in Figure 6. Within 0~90 min, the starch hydrolysis rate increased rapidly, and after 90 min, it showed a slow rise and gradually leveled off. The starch hydrolysis rates of MLE prepared by different drying methods were lower than those of the control group at various time points. The change curves of HADMLE and SDMLE were similar, but FDMLE exhibited a smaller variation in starch hydrolysis rate within 120 min. At 180 min, there was no significant difference between HADMLE and the control group, but FDMLE and SDMLE showed significant reductions. It was speculated that MLE can act as a carbohydrate digestive enzyme inhibitor, possibly by competitively binding to the active sites of digestive enzymes with the substrate starch, thereby slowing down the starch digestion rate [55]. Among them, FDMLE exhibited the most significant inhibitory effect on starch digestion (*p* < 0.05). Liu et al. [10] investigated the effects of different drying methods on the phenolic components and in vitro hypoglycemic activity of pulp extracts from two bayberry cultivars, and demonstrated that freeze-dried samples possessed significantly higher TFC and TAC, and exhibited more potent antioxidant and hypoglycemic activities than hot-air-dried samples (*p* < 0.05). Inhibition of the starch digestion activity is found to be effective to decrease the postprandial hyperglycemia, and it is regarded as an important treatment strategy in type II diabetes. Meanwhile, combined with Table 2, it is speculated that flavonoids are the main contributors to inhibiting starch digestion of mulberry leaf extract.

## 4. Conclusions

This study demonstrated that drying methods significantly influence the bioactive compounds, VOCs, and functional properties of MLEs. FDMLE exhibited the highest TFC and retained the ethanol flavor, while SD improved color stability and generated unique aldehydes and ketones, imparting a grassy aroma. However, HAD reduced TFC due to prolonged high-temperature exposure, though the presence of ethyl formate, rose oxide, and (Z)-3-hexenol in HADMLE contributed to distinctive rose and citrus notes. Notably, FDMLE showed the strongest inhibition of starch digestion in vitro, highlighting its potential as a high-quality drying method to prevent degradation of MLEs. These findings provide novel evaluation criteria and theoretical insights for optimizing drying processes to maximize MLE product quality. Although this study developed various drying methods for MLE, the scalability constraints caused by the prolonged processing time and high energy consumption of FD remain critical bottlenecks for industrial adoption. Therefore, the development of innovative technologies such as microwave-assisted FD to mitigate the energy demands and operational expenses of conventional FD, while preserving the quality attributes of MLE, represents a critical imperative for future research.

## Figures and Tables

**Table 1:** WC, TA, and TFC of FDMLE, HADMLE, SDMLE, and ML.

	FDMLE	HADMLE	SDMLE
WC (%)	17.35 ± 0.12 ^a^	13.22 ± 0.07 ^b^	12.09 ± 0.53 ^b^
TAC (mg DNJ/g DW)	0.14 ± 0.02 ^a^	0.13 ± 0.01 ^a^	0.13 ± 0.02 ^a^
TFC (mg RE/g DW)	19.32 ± 0.58 ^a^	4.89 ± 0.30 ^c^	13.70 ± 0.18 ^b^

Notes: Each value in the table is the mean ± standard deviation (*n* = 3). Different superscript letters in the same row indicate statistical significance (*p* < 0.05).

**Table 2:** Color values of FDMLE, HADMLE, SDMLE, and ML.

Sample	L*	a*	b*	Δ*E*
FDMLE	4.67 ± 0.01 ^b^	−2.69 ± 0.01 ^b^	1.66 ± 0.00 ^b^	1.76 ± 0.00 ^b^
HADMLE	5.61 ± 0.29 ^a^	−2.65 ± 0.03 ^b^	2.94 ± 0.01 ^a^	3.28 ± 0.01 ^a^
SDMLE	4.16 ± 0.03 ^c^	−2.72 ± 0.06 ^b^	1.55 ± 0.09 ^b^	1.55 ± 0.04 ^c^
ML	4.16 ± 0.03 ^c^	−2.41 ± 0.03 ^a^	1.63 ± 0.05 ^b^	

Notes: Each value in the table is the mean ± standard deviation (*n* = 3). Different superscript letters in the same column indicate statistical significance (*p* < 0.05).

**Table 3:** The information on detected VOCs of MLE treated by different methods by GC-IMS.

Count	Compound	CAS#	Formula	*m*_w_	RI	Rt (s)	Dt [a.u.]
Aldehydes							
1	butanal	C123728	C_4_H_8_O	72.1	860.1	275.73	1.11252
2	3-Methyl-2-butenal (D)	C107868	C_5_H_8_O	84.1	1214.2	671.019	1.09273
3	3-Methyl-2-butenal (M)	C107868	C_5_H_8_O	84.1	1214.2	671.019	1.36005
4	(E)-2-hexenal (D)	C6728263	C_6_H_10_O	98.1	1234.3	698.712	1.51411
5	pentanal	C110623	C_5_H_10_O	86.1	967.8	342.119	1.185
6	nonanal	C124196	C_9_H_18_O	142.2	1403.3	983.026	1.4784
7	(E)-2-hexenal (M)	C6728263	C_6_H_10_O	98.1	1234.9	699.635	1.18354
8	methacrolein	C78853	C_4_H_6_O	70.1	901.8	299.743	1.22033
9	Heptal dehyde (M)	C111717	C_7_H_14_O	114.2	1196.1	647.138	1.3358
10	Heptaldehyde (D)	C111717	C_7_H_14_O	114.2	1195.8	646.728	1.6909
11	Hexanal	C66251	C_6_H_12_O	100.2	1104.5	486.272	1.26628
12	(Z)-4-heptenal	C6728310	C_7_H_12_O	112.2	1256.1	730.033	1.14792
13	2-Methylbutanal	C96173	C_5_H_10_O	86.1	931.5	318.106	1.40064
14	Furfural (D)	C98011	C_5_H_4_O_2_	96.1	1490.9	1173.81	1.33121
15	Furfural (M)	C98011	C_5_H_4_O_2_	96.1	1493	1179.022	1.08437
Alcohols							
16	(Z)-3-hexenol (D)	C928961	C_6_H_12_O	100.2	1400	976.564	1.51207
17	(Z)-3-hexenol (M)	C928961	C_6_H_12_O	100.2	1401.4	979.334	1.22843
18	2-hexanol (D)	C626937	C_6_H_14_O	102.2	1218.5	676.877	1.27363
19	2-hexanol (M)	C626937	C_6_H_14_O	102.2	1433.5	1045.142	1.19265
20	3-Methyl-3-buten-1-ol	C763326	C_5_H_10_O	86.1	1230.8	693.845	1.43605
21	ethanol	C64175	C_2_H_6_O	46.1	948.4	329.053	1.12792
22	2-Methyl-1-propanol (D)	C78831	C_4_H_10_O	74.1	1105.3	487.503	1.36492
23	2-Methyl-1-propanol (M)	C78831	C_4_H_10_O	74.1	1106.9	488.674	1.41296
24	butan-1-ol (D)	C71363	C_4_H_10_O	74.1	1156.9	574.092	1.38183
25	butan-1-ol (M)	C71363	C_4_H_10_O	74.1	1157.1	574.502	1.18173
26	3-Methyl-1-butanol	C123513	C_5_H_12_O	88.1	1218.9	677.506	1.48235
27	2-Methyl-1-butanol	C137326	C_5_H_12_O	88.1	1219.2	677.916	1.2334
28	Propan-1-ol (D)	C71238	C_3_H_8_O	60.1	1051	419.456	1.25839
29	Propan-1-ol(M)	C71238	C_3_H_8_O	60.1	1053.8	422.634	1.1098
30	Butan-2-ol	C78922	C_4_H_10_O	74.1	1035.1	401.799	1.14876
31	1-Hexanol	C111273	C_6_H_14_O	102.17	1433.5	1045.142	1.19265
32	3-methylthiopropanol	C505102	C_4_H_10_OS	106.2	1701.7	1798.795	1.08406
Ketones							
33	6-methylhept-5-en-2-one	C110930	C_8_H_14_O	126.2	1347.9	878.716	1.17639
34	1-penten-3-one	C1629589	C_5_H_8_O	84.1	1040.9	408.155	1.31094
35	2-pentanone (D)	C107879	C_5_H_10_O	86.1	976.5	348.123	1.39429
36	2-pentanone (M)	C107879	C_5_H_10_O	86.1	978.1	349.182	1.12611
37	Butan-2-one	C78933	C_4_H_8_O	72.1	919.7	310.69	1.24208
38	1-Hydroxy-2-propanone	C116096	C_3_H_6_O_2_	74.1	1317.9	827.023	1.24373
39	4-Methyl-2-pentanone	C108101	C_6_H_12_O	100.2	994.5	360.835	1.47946
40	3-Hydroxy-2-butanone	C513860	C_4_H_8_O_2_	88.1	1301.1	799.33	1.06416
Esters							
41	ethyl acetate (D)	C141786	C_4_H_8_O_2_	88.1	903.5	300.803	1.3345
42	ethyl acetate (M)	C141786	C_4_H_8_O_2_	88.1	904.1	311.604	1.1578
43	Ethyl formate	C109944	C_3_H_6_O_2_	74.1	836	262.752	1.06638
44	ethyl-2-methyl butanoate	C7452791	C_7_H_14_O_2_	130.2	1035.6	402.305	1.22615
45	butyl butanoate	C109217	C_8_H_16_O_2_	144.2	1232.9	696.767	1.33524
46	2-methylpropyl acetate	C110190	C_6_H_12_O_2_	116.2	1018.2	383.795	1.23448
47	methyl butanoate	C623427	C_5_H_10_O_2_	102.1	975.5	347.416	1.4405
48	methyl 2-methylbutanoate	C868575	C_6_H_12_O_2_	116.2	1009.2	374.608	1.19044
Others							
49	2(3H)-Furanone, 5-methyl(D)	C591128	C_5_H_6_O_2_	98.1	1442	1063.336	1.12436
50	2(3H)-Furanone, 5-methyl(M)	C591128	C_5_H_6_O_2_	98.1	1442.5	1064.259	1.38352
51	2-acetylfuran (D)	C1192627	C_6_H_6_O_2_	110.1	1536.9	1288.487	1.1239
52	2-acetylfuran (M)	C1192627	C_6_H_6_O_2_	110.1	1538.9	1293.7	1.44102
53	2-acetylthiazole (D)	C24295032	C_5_H_5_NOS	127.2	1644.4	1601.842	1.12752
54	2-acetylthiazole (M)	C24295032	C_5_H_5_NOS	127.2	1645.5	1605.278	1.47801
55	rose oxide	C16409431	C_10_H_18_O	154.2	1107.6	491.196	1.17422
56	1,4-dioxane	C123911	C_4_H_8_O_2_	88.1	1084.8	459.598	1.12819
57	2,6-Dimethylpyrazine	C108509	C_6_H_8_N_2_	108.1	1359.2	899.088	1.1393
58	2,5-dimethyl thiophene	C638028	C_6_H_8_S	112.2	1192.9	642.895	1.08013
59	Acetic acid (D)	C64197	C_2_H_4_O_2_	60.1	1495.9	1185.973	1.16167
60	Acetic acid (M)	C64197	C_2_H_4_O_2_	60.1	1504.6	1206.823	1.05187
61	Ethylbenzene	C100414	C_8_H_10_	106.2	1142.1	547.828	1.07746
62	N, N-dimethylacetamide	C127195	C_4_H_9_NO	87.1	1410.2	996.872	1.06824
63	3-carene	C13466789	C_10_H_16_	136.2	1131	528.951	1.21367

## Data Availability

The original contributions presented in this study are included in the article. Further inquiries can be directed to the corresponding authors.

## Abbreviations

The following abbreviations are used in this manuscript:

ML	mulberry leave
MLE	mulberry leaf extracts
FD	freeze-drying
HAD	hot air drying
SD	spray drying
TAC	total alkaloids content
TFC	total flavonoid content
VOC	volatile compound
GC-IMS	gas chromatography-ion mobility spectrometry
SEM	scanning electron microscopy
